# Development of Environmentally Friendly and Economical Flood-Prevention Stones Based on the Sediments of the Yellow River

**DOI:** 10.3390/gels10100622

**Published:** 2024-09-27

**Authors:** Ying Liu, Hao Xiao, Yongxiang Jia, Yajun Lv, Li Dai, Chen Yang

**Affiliations:** 1 College of Water Resources, North China University of Water Resources and Electric Power, Zhengzhou 450046, China; 2 Collaborative Innovation Center for Efficient Utilization of Water Resources, North China University of Water Resources and Electric Power, Zhengzhou 450046, China; 3 Henan ZhongGong Design & Research Group Co., Ltd., Zhengzhou 450046, China; 4 College of Architecture, North China University of Water Resources and Electric Power, Zhengzhou 450046, China; 5 School of Environmental and Municipal Engineering, North China University of Water Resources and Electric Power, Zhengzhou 450046, China

**Keywords:** Yellow River sediment, flood-prevention stones, alkali excitation, C-A-S-H gel, environmentally friendliness

## Abstract

The deposition of Yellow River sediment in the middle and lower reaches is a significant factor in the siltation of reservoirs and the occurrence of serious flooding along the river. The efficient and valuable utilization of Yellow River sediment has already become a key research topic in this field. In this study, we have employed Yellow River sediment as the primary material, in conjunction with commercially available slag, fly ash, and quicklime as the binder, to develop a novel type of artificial flood-prevention stone. Following a 28-day standard curing procedure, the highest compressive strength of the prepared artificial stone was recorded at 4.29 MPa, with a value exceeding 0.7 MPa under wet conditions. The results demonstrated that the prepared artificial stone met the specifications for artificial flood-prevention stones. The curing mechanism, as evidenced by analyses from SEM and XRD testing, indicated that the alkali excitation process in the binder, which produced C-A-S-H gel, was the key factor in enhancing the compressive strength of the specimens. Notably, an evaluation of the amount of CO_2_ emissions and the cost of the artificial stone concluded that the preparation process was both environmentally friendly and cost-effective.

## 1. Introduction

The Yellow River is a prototypical sediment-laden river, exhibiting the highest sediment load in the world [1,2]. As the river slows down in the middle and lower reaches, it gradually accumulates sediment, depositing more than 400 million cubic meters of sediment each year, making it vulnerable to various hydraulic disasters [3,4,5,6]. The occurrence of floods and other hydraulic disasters in the middle and lower reaches of the Yellow River has resulted in significant damage and has constituted a major constraint on China’s socio-economic development [7,8,9,10]. In order to mitigate the impact of these disasters, management strategies have been implemented for the Yellow River. As one of the key management strategies, significant quantities of flood-prevention stones, characterized by their specific volume and weight, have been strategically positioned in crucial locations such as perilous bends, checkpoints, and culverts along the middle and lower sections of the river [11,12,13,14,15]. It is estimated that the current annual consumption of stone for flood prevention is 600,000 cubic meters. As China’s economic development model has shifted to prioritize environmental protection, the extraction of non-renewable natural stone for flood prevention has declined [16,17]. Therefore, facile preparation methods of artificial flood-prevention stones would effectively address this deficit.

The utilization of alkali activation for converting silt into cementitious materials has been well documented by numerous research teams. This process leverages the catalytic effect of alkaline activators to expedite the hydration process and enhance the hydration reaction rate [18,19,20]. This applied technology is presently employed across various engineering applications, including the resource utilization of Yellow River sediment [21,22,23]. For instance, Deng and co-workers, in 2022, presented a preparation scheme for ultra-high-performance concrete (UHPC) from ultra-fine sand sourced from the Yellow River [22]. The scheme was developed using the alkali activation principle and discussed the probability of transporting sediment from the bottom of the reservoir to the shore and building an embankment on both sides of the reservoir. 

In comparison, the number of studies conducted on the utilization of Yellow River sediment for the preparation of artificial flood-prevention stones is relatively limited. Wang, in 2017, used the Yellow River sediment alkali activation method to produce a kind of artificial flood-prevention stone, and studied the effects of the amount of alkali, slag content, and curing time on the compressive strength of artificial flood-prevention stones, and the test block made with the optimal ratio could meet the requirements of flood-prevention stones [24]. Zheng and co-worker modified Yellow River sediment by alkali excitation, and added granulated blast furnace slag powder, red coal slime, black coal slime, and other admixtures to prepare flood-prevention stones with a 90d compressive strength greater than 10 MPa [25]. 

While instances of preparing artificial flood-prevention stones have been previously mentioned, it is imperative to acknowledge the limitations of previous studies. Firstly, the alkaline activation method typically requires the addition of alkaline compounds, such as Ca(OH)_2_ or NaOH, as activators in a mass ratio of at least 5% to produce specimens with high compressive strength. However, the presence of these alkaline substances in the stones placed in water can significantly increase the pH of the water, which can have irreversible negative impacts on the environment and its corresponding organisms. Secondly, the financial implications of the preparation of artificial flood-prevention stones have been omitted from the aforementioned works. We hypothesize that the use of specific industrial by-products in place of the alkaline activators mentioned above has the potential to significantly reduce the cost of stone preparation, thereby increasing the viability of producing and marketing artificial flood-prevention stones.

In light of the aforementioned research, and the shortcomings in the previous studies, we herein present the synthesis of a novel artificial flood-prevention stone. The optimal amount of each component of the binder was preliminarily determined through the use of an orthogonal experimental design and simulation calculations by Design-Expert. Moreover, the mechanical properties of the prepared specimens and the pH of the leaching solution were evaluated, and it was determined that the prepared specimens satisfied the criteria for utilization as artificial flood-prevention stones. The microstructure of the samples was analyzed, and it was concluded that the hydrogel reaction between calcium oxide in slag and reactive silicon dioxide and aluminum oxide in fly ash to form C-A-S-H gel was the dominant reason for the increase in the compressive strength of the specimens. It is noteworthy that the calculation of CO_2_ emissions from the preparation process and the cost of preparation indicates that the production process of the designed stones is environmentally friendly. The lower production cost is favorable for the further promotion of the stones on the market.

## 2. Materials and Methods

### 2.1. Materials

**Figure 1:**
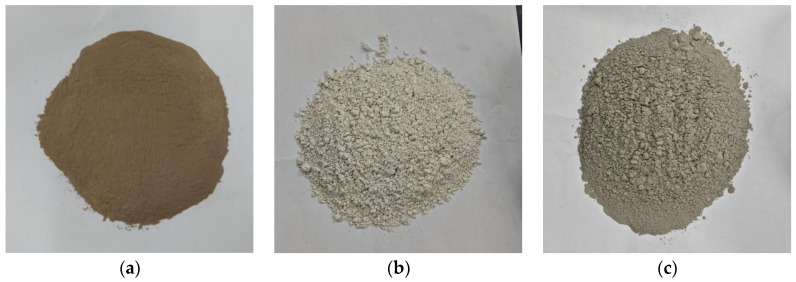
Appearance of Yellow River sediment (**a**), slag (**b**), and fly ash (**c**).

**Figure 2:**
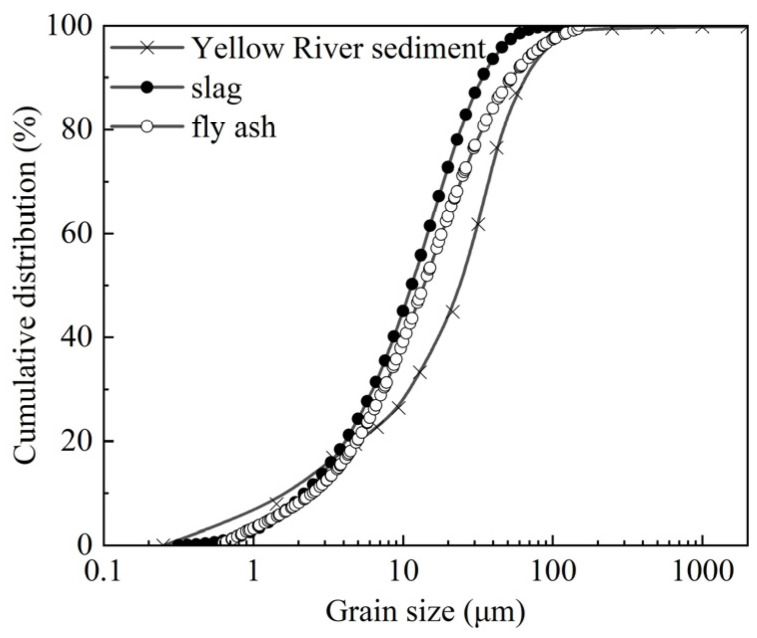
Gradation curves of Yellow River sediment, slag, and fly ash in this work.

The sediment from the Yellow River, used in this work, was sourced from the dredged deposits of the Sanmenxia Reservoir in the Henan Province. Prior to the curing procedure, the sediment was subjected to drying in an oven maintained at a temperature of 105 °C for a period of 24 h. The slag employed in the treatment of the sediment was the by-product of ore crushing and processing, procured in Gongyi City. The fly ash was produced by the Shandong Rongchangsheng Environmental Protection Materials Factory. The visual characteristics and the outcomes of the particle size distribution tests for the raw materials are illustrated in Figure 1 and Figure 2, respectively.

Samples for XRF analysis were prepared according to the following procedure: The selected blocks were dried at 105 °C until a constant weight was reached. The materials were then crushed, and the resulting powder was sieved through an 80 μm sieve. The mass of the samples tested was at least 3 g. The aforementioned materials were subjected to a composition analysis through the utilization of X-ray fluorescence spectrometry (XRF) (Panalytical Axios, PANalytical B.V., Almelo, Netherlands), with the findings presented in Table 1. Furthermore, the quicklime that was employed as one of the selected binders was identified as dolomitic lime, with a calcium oxide (CaO) content of 70.42% and a magnesium oxide (MgO) content of 10.51%.

### 2.2. Sample Preparation

The dried Yellow River sediment and designed binder mixture were placed in an electrical agitator, followed by the addition of water with a mass fraction of 25%. Subsequently, the mixture was compacted into 40 mm × 40 mm × 40 mm specimens by compression molding. In order to ensure a compacted density of 1.78 g/cm^3^, a minimum pressure of 2.33 MPa was applied during the molding process. Following a 24 h period of room temperature curing, the specimens were transferred to a standard curing box (20 ± 2 °C, 95% RH) for a further period of curing. The curing durations were set at 7 and 28 days.

The binder employed in this study was a combination of slag, fly ash, and quicklime. In order to ascertain the optimal binder formulation, a multifactorial test was designed, which incorporated the aforementioned three materials. In the binders, the proportions of slag and fly ash were varied at three levels: 2%, 5%, and 8%, with quicklime serving as the complementary component. The specific quantities of each substance in the binder are delineated in Table 2. It is notable that the dosage of the binder constitutes 20% of the combined mass of the specimen.

Recently, Song investigated the protocol for the production of concrete by alkali excitation using fly ash and sodium silicate [26]. The results showed that an appropriate amount of quicklime promoted the formation of N-A-S-H and C-S-A gels, thereby increasing the compressive strength of the specimens. In order to guarantee the progression of alkali activation in our research, the minimum dosage of quicklime in the binder was established at 20%, representing 4% of the total specimen mass. To ensure the optimal mix ratio of the binder, a single-factor influence experiment was conducted, wherein the quantity of slag was systematically varied to replace the fly ash continuously. The experimental design is outlined in Table 3.

### 2.3. General Testing Methods

To characterize the strength of the specimens for Yellow River sediment-based flood-prevention stones, unconfined compressive strength tests (UCS) were carried out in accordance with ASTM D4219. For the purpose of pH testing the leaching solution, the specimens were crushed and then sieved through a square sieve with a mesh size of 0.08 mm. Subsequently, the specimens were combined with water in a 1:10 ratio for the purpose of pH testing, in accordance with standard ASTM D4972-01. The freeze–thaw resistance of the prepared specimens, which were cured for 28 days, was examined through a series of freeze–thaw cycles, with intervals of 2, 5, 10, and 20 days [27]. The softening coefficient of the specimens, designated as flood-prevention stone and cured for a duration of 28 days [28], was calculated with the below equation.

$$softening\;coefficent=\frac{wetting\;strength\;(MPa)}{drying\;strength\;(Mpa)}$$

Microscopic morphology testing of samples under different ratios was conducted using a scanning electron microscope (SEM) (MIRA LMS, Tescan, Brno-Kohoutovice, Czech Republic) at resolutions set to 3 nm. Testing samples were magnified at 500 and 5000 times (20 µm and 2 µm). The acceleration voltage ranged from 0.5 kV to 30 kV, with low vacuum pressure set between 1 Pa and 270 Pa. Additionally, the samples cured for 7 and 28 days under different mix ratios were tested using an X-ray diffractometer (XRD) (Rigaku Ultima IV X, Rigaku, Japan). The scanning angle range was set from 5° to 80° at a scanning rate of 20°/min, with a wavelength of 1.5418, voltage of 40 kV, and current of 40 mA. Powder from specimens passing through an 80 µm sieve was used for testing.

## 3. Results and Discussion

### 3.1. Analysis of Multi-Factor Compressive Strength Test Results

**Figure 3:**
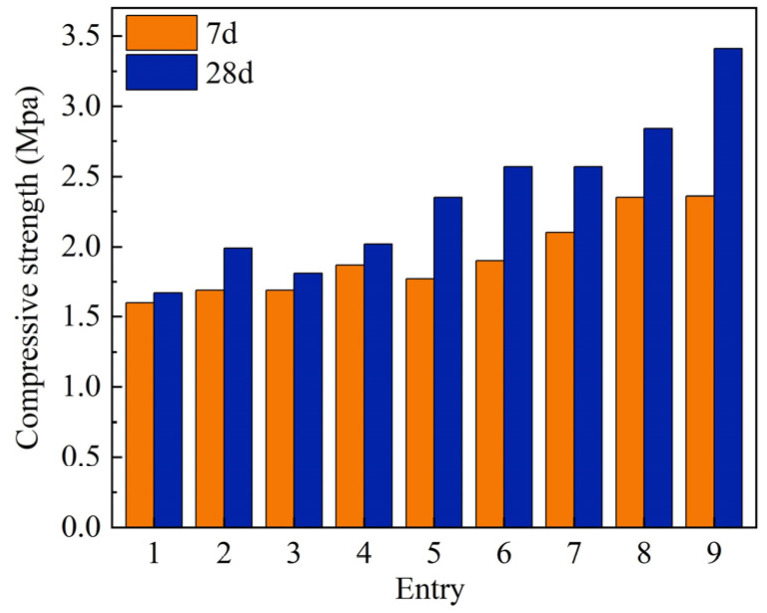
Compressive strength test results of multi-factor experiments.

The outcomes of the compressive strength assessments of the prepared specimens under the conditions of varying binder formulations (Table 2) are illustrated in Figure 3. In general, the compressive strength index of the specimens exhibited a notable enhancement following a 28-day curing period, in comparison to the 7-day curing period. The formulae for the compressive strength test results are presented as follows:

Compressive strength (MPa, 7d) = 3.35 × *A* + 1.73 × *B* + 1.31 × *C*

Compressive strength (MPa, 28d) = 4.82 × *A* + 0.13 × *B* + 1.52 × *C* + 7.04 × *AB* − 3.04 × *AC* + 2.3 × *BC*

*A:* dosage of slag (%); *B*: dosage of fly ash (%); *C*: dosage of quicklime (%). The regression coefficients were calculated by a linear regression analysis with interaction using Design-Expert. 

Notably, the compressive strength of the specimens derived from the various binder formulations exhibited significant variation. The maximum compressive strengths of 2.36 MPa and 3.41 MPa were achieved at 7 and 28 days of curing, respectively, using a binder comprising 40% slag, 40% fly ash, and 20% quicklime (Table 2, entry 9). Furthermore, the data presented in Figure 3 suggest that reducing the quantity of quicklime and increasing the proportion of slag resulted in an improvement in the compressive strength of the specimens.

**Figure 4:**
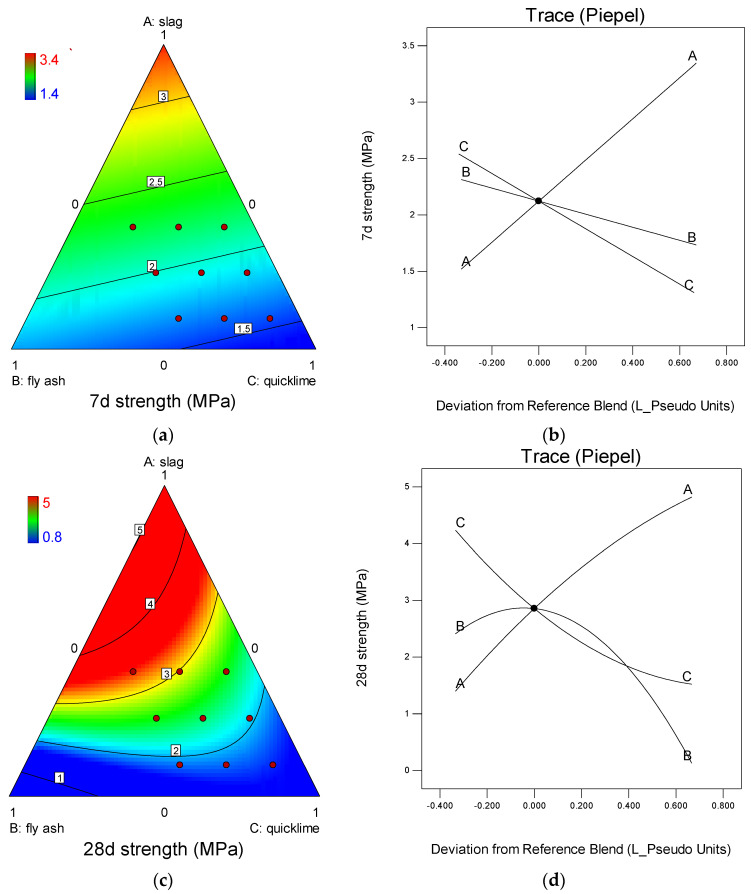
Analysis of the compressive strength of the prepared flood−prevention stone. (**a**) Ternary plot of 7−day compressive strength; (**b**) single−factor influence analysis of 7−day compressive strength; (**c**) ternary plot of 28−day compressive strength; (**d**) single−factor influence analysis of 28−day compressive strength.

To accurately illustrate the influence of the binder components on the compressive strength test outcomes, a simulation analysis was conducted using Design-Expert (Figure 4). The compressive strength of Yellow River sediment, cured for seven days, demonstrated a nearly linear relationship with variations in the slag, fly ash, and quicklime (Figure 4a). The highest compressive strength was observed when the slag content was at 100%, indicating that the strength of the cured Yellow River sediment at 7 days was predominantly influenced by the slag content. Figure 4b of the compressive strength analysis reveals that the compressive strength of the cured Yellow River sediment at 7 days increased with an increase in the slag content but decreased with an increase in the fly ash and quicklime content. Indeed, an increase in the proportion of slag in the binder resulted in a corresponding rise in the levels of reactive silicon dioxide (SiO_2_) and aluminum oxide (Al_2_O_3_), which in turn led to an enhancement in the strength of the cured Yellow River sediment. Consequently, the slag within the binder was identified as the primary component responsible for conferring early strength to the solidified Yellow River sediment.

The compressive strength results after a 28-day curing period exhibited a distinct trend (Figure 4c) when compared to the observations made after a 7-day curing period. The analysis indicated that the optimal compressive strength of the specimen, at 4.31 MPa, was achieved with a dosing composition of 68% slag and 32% fly ash, in the absence of quicklime. An initial increase in the compressive strength was observed as slag replaced fly ash, followed by a subsequent decrease (Figure 4d). Meanwhile, the incorporation of quicklime had a negative effect on the compressive strength during the specified curing duration.

The results of the aforementioned analyses indicated that the primary contribution of slag to the compressive strength of Yellow River sediment was during its initial curing process. During the extended curing period, the inclusion of fly ash in addition to the compressive strength provided by the slag led to a subsequent enhancement in the compressive strength of the specimens. However, it is noteworthy that while the inclusion of quicklime slightly decreased the compressive strength of the specimens, a 20% quicklime supplement was incorporated as a binder in the following curing experiments to ensure the initiation of the alkaline excitation process.

It is notable from the data that the minimum compressive strength achieved after a curing period of 28 days was merely 0.8 MPa, as depicted in Figure 4c. This value is considerably lower than the minimum compressive strength of 1.4 MPa observed after just 7 days of curing, as shown in Figure 4a. The discrepancy in these results can be directly attributed to the compositional differences in the binder used. Specifically, the binder utilized in the specimens that underwent 28 days of curing consisted entirely of fly ash. Referring to Table 2, it becomes evident that the chosen fly ash composition possesses an exceptionally low content of reactive CaO. This characteristic impedes its ability to effectively react with reactive SiO_2_ and Al_2_O₃. Consequently, under such conditions, the primary contributor to the compressive strength of the specimens is the internal consolidation process.

### 3.2. The Influence of Slag Content on Macroscopic Performance

#### 3.2.1. Compressive Strength Testing

**Figure 5:**
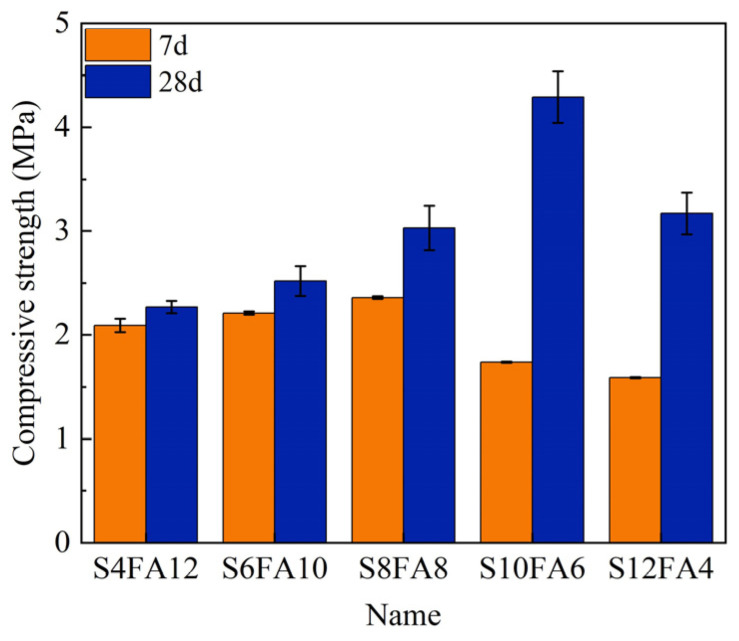
Compression strength results with stepwise slag and fly ash ratios.

Upon establishing that 4% of the total weight comprised quicklime, an investigation was conducted into the impact of varying doses of slag and fly ash on the compressive strength of Yellow River sediment flood-prevention stones (Table 3). In accordance with the preceding research results, the compressive strength was determined for the conditions of 7 and 28 days of curing time, respectively, and the results are presented in Figure 5. 

In general, an increase in the curing duration resulted in a positive impact on the compressive strength of the specimens. The results presented in Figure 5 demonstrated that a prolonged curing time, in conjunction with an elevated slag content in the binder, resulted in a notable enhancement in the compressive strength. For example, the addition of 10% slag and 6% fly ash to the binder (S10FA6) resulted in a 146% increase in the compressive strength after 28 days of curing compared to the same curing conditions with a 7-day curing time.

A 7-day curing period resulted in the activation of SiO_2_ and Al_2_O_3_ in the fly ash, which was evident from the formation of a C-A-S-H gel with enhanced compressive strength in the specimens. The highest compressive strength index was observed for specimens with a slag dosage of 8% (S8FA8). Nevertheless, an increase in the dosage of slag resulted in a reduction in the compressive strength of the specimens. It was hypothesized that the excess CaO in the slag was unable to form an effective C-A-S-H gel, leading to the downward trend.

Conversely, the specimens cured for 28 days with a composition of 10% slag and 6% fly ash exhibited the highest compressive strength, reaching 4.29 MPa. These findings were in accordance with the results of our prior analysis (Figure 4c). The observed enhancement in the compressive strength can be attributed to two primary factors. Firstly, the optimal ratio of CaO from slag and the activation of SiO_2_ and Al_2_O_3_ from fly ash in the formation of C-A-S-H gel within the binder. Secondly, the extension of the curing period facilitated additional growth of the C-A-S-H gel, thereby bolstering the compressive strength of the specimens. Our presumption in this section was further proved by subsequent microscopic structure analyses.

#### 3.2.2. Leaching Solution pH Testing

**Figure 6:**
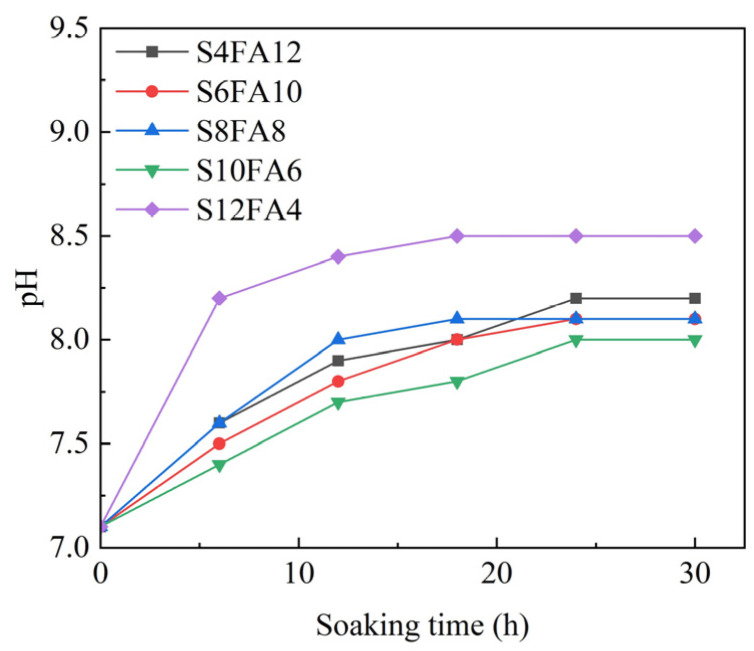
pH of leaching solution.

The components of Yellow River sediment flood-prevention stones, particularly CaO, which is the main constituent of slag and quicklime, exert a considerable influence on the pH of the water during long-term immersion. Herein, we examined the pH of the leachate in the case of different durations of immersion in water of the specimens after 28 days of curing, and the results are presented in Figure 6.

The test results demonstrated that specimens containing less than 10% slag content within the binder exhibited a leachate pH of below 8.2 after being immersed for 30 h. It was hypothesized that the presence of CaO in slag and quicklime facilitated an effective combination with active SiO_2_ and Al_2_O_3_ in fly ash, resulting in the production of C-A-S-H gel, which was responsible for enhancing the compressive strength as previously discussed. It is notable that an increase in the slag content to 12% resulted in a reduction in the quantity of active substances presented in the fly ash, thereby enabling the excess CaO to interact with water and form alkaline Ca(OH)_2_ in a process that resulted in a marked increase in the pH of the specimens during the initial period of immersion, with the final pH maintained at 8.5. Notably, compared to the leaching solution pH outcomes for biomass polymer lignin-stabilized silt (with a final pH reaching 10.0) [29] and cement-stabilized soils (exceeding a final pH of 12) [30], the artificial flood-prevention stone produced in our study appears to be environmentally benign.

#### 3.2.3. Rapid Freeze–Thaw Cycling Testing

**Figure 7:**
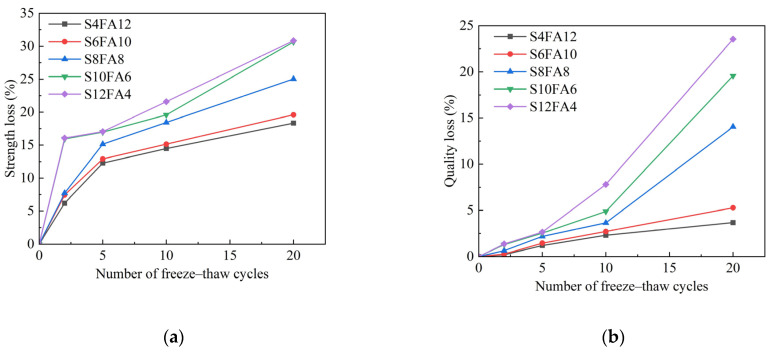
Effect of rapid freeze–thaw cycle on the strength (**a**) and quality (**b**) of flood-prevention stones. (**a**) Compressive strength loss in rapid freeze–thaw cycling; (**b**) quality loss in rapid freeze–thaw cycling.

A rapid freeze–thaw cycle test was conducted to evaluate the influence of the freeze–thaw process on the prepared specimens, particularly in regard to their loss of compressive strength and mass. The corresponding test results are presented in Figure 7.

In the initial five freeze–thaw cycles, a notable decline in the compressive strength was observed for the specimens (Figure 7a). The rate of loss of compressive strength in the specimens exhibited a progressive deceleration with an increase in the number of freeze–thaw cycles. It is noteworthy that an increase in the proportion of fly ash in the binder resulted in a reduction in the rate of strength degradation and an improvement in the stone’s durability. In the case study involving a 10% supplementation of fly ash to the test block (S6FA10), the reduction in compressive strength remained below 20% following 20 freeze–thaw cycles. It is pleasing to report that the prepared specimens have met the specified requirements for artificial flood-prevention stones.

Similarly, the quality of the prepared stone specimens was found to be adversely affected by rapid freeze–thaw cycling. As the frequency of freeze–thaw cycles increased, a reduction in the compressive strength was accompanied by a clear decrease in the quality of the specimens (Figure 7b). Fortunately, after 10 freeze–thaw cycles, the majority of specimen blocks exhibited a mass loss of less than 5%, which is lower than the similar work by Gutiérrez et al, in 2020, who declaim a 7.45% loss of mass [31], thereby satisfying the requisite specifications for artificial flood-prevention stones. 

It is plausible to posit that the formation of a C-A-S-H gel plays a crucial role in preserving the compressive strength and mass of specimens during rapid freeze–thaw cycling. This is evident from the results depicted in Figure 7, which show that an increased dosage of fly ash in the binder leads to a negligible increase in the mass loss of specimens as the number of freeze–thaw cycles escalates. We hypothesized that augmenting the mass fraction of fly ash, rich in active SiO_2_ and Al_2_O_3_ components, in the binder, along with an additional 4% mass fraction of quicklime, facilitated the generation of C-A-S-H gel. Conversely, in specimens with elevated slag content, such as S12A4 and S10A6, surplus active CaO failed to effectively form C-A-S-H gel due to limited active SiO_2_ and Al_2_O_3_. We also anticipated that the production of Ca(OH)_2_ and following CaCO_3_ from excess CaO during rapid freeze–thaw cycling did not fulfill the requirements for the compressive strength and quality of the specimens.

#### 3.2.4. Softening Coefficient Testing

**Figure 8:**
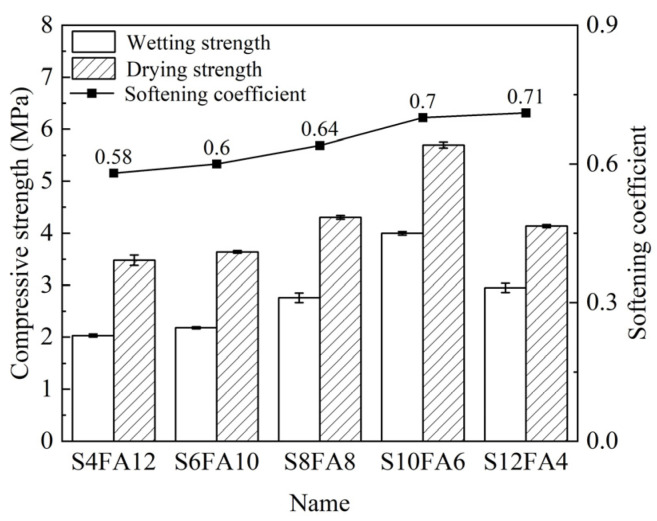
Softening coefficient of Yellow River sediment-based flood-prevention stones.

In order to guarantee the maintenance of robust strength following an extended period of immersion, a series of experiments were conducted with the objective of determining the softening coefficient of the flood-prevention stones that were presented (Figure 8). In general, the compressive strength of the prepared specimens was analyzed under conditions of moisture. The highest compressive strength was observed in both dry and wet conditions for the test samples that contained 10% slag content (SA10F6).

Notably, the specimens exhibiting a slag content in the binder in excess of 10% displayed a softening coefficient of at least 0.7. Prior analyses determined that an amount of C-A-S-H gel, which enhanced the compressive strength, was sufficient to be formed after 28 days of curing. The creation of the gel maintained the compressive strength of the specimen in wet conditions. 

### 3.3. Effect of Slag Content Variation on Microstructure

#### 3.3.1. Mechanism Analysis

Our findings indicate that the formation of a C-A-S-H gel, resulting from the reaction between CaO in slag and active SiO_2_ and Al_2_O_3_ in fly ash, is a necessary condition for the specimens to exhibit the desired properties and to meet the specifications for artificial flood-prevention stones. The potential reaction equations are presented below:

Firstly, CaO reacted with water to form Ca(OH)_2_, as shown in the following chemical equation:

$$CaO+H_{2}O\to {Ca(OH)}_{2}$$

The hydration process of slag entailed a pozzolanic reaction involving active SiO_2_, Al_2_O_3_, and Ca(OH)_2_ within the slag, leading to the creation of C-S-H and C-A-H gels, which were recognized as the primary source of compressive strength for the prepared Yellow River sediment flood-prevention stones in this work. The key reaction is demonstrated as follows:

$$Active\;SiO_{2}+m_{1}Ca{\left(OH\right)}_{2}+nH_{2}O\to m_{1}CaO\cdot SiO_{2}\cdot nH_{2}O$$

$$Active\;{Al}_{2}O_{3}+m_{2}Ca{\left(OH\right)}_{2}+nH_{2}O\to m_{2}CaO\cdot {Al}_{2}O_{3}\cdot nH_{2}O$$

#### 3.3.2. Microscopic Structure

**Figure 9:**
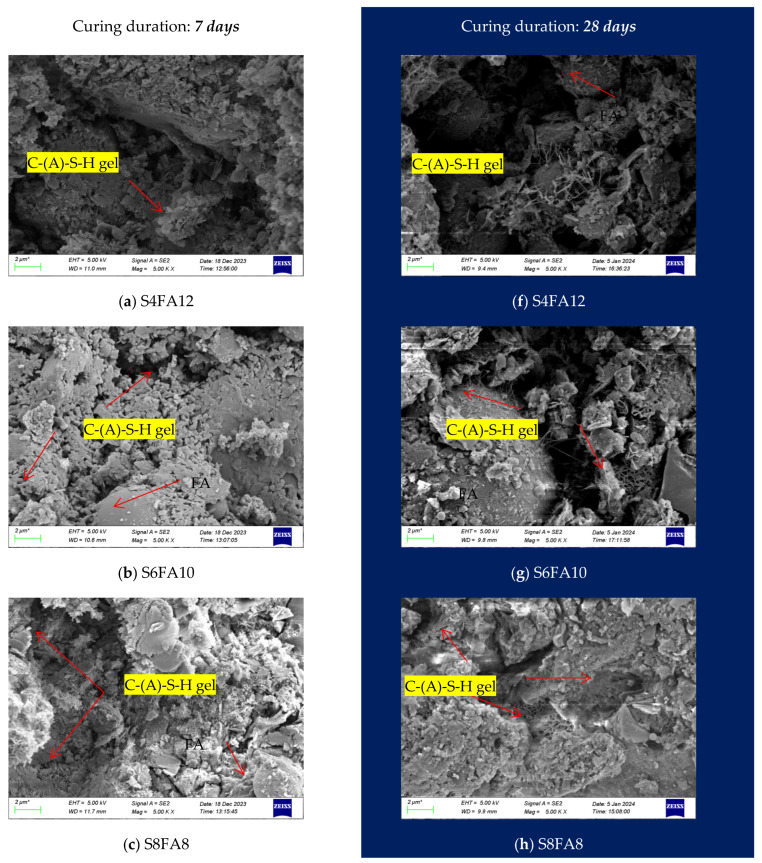

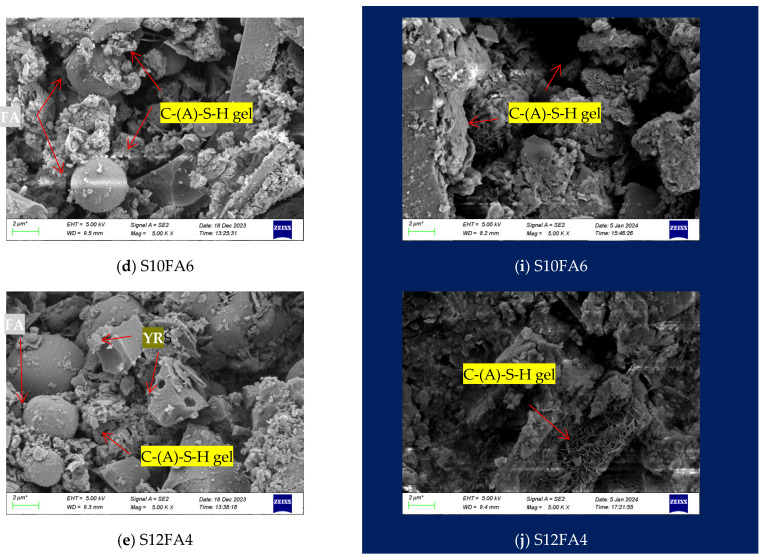
SEM diagram of prepared flood-prevention stones at 7 and 28 days of curing. FA: fly ash; YRS: Yellow River Sediment.

Figure 9 illustrates the scanning electron microscope (SEM) images of the Yellow River sediment-based flood-prevention stones under different binders. A comparison of the SEM images of samples cured for 7 and 28 days under identical binders revealed that the structural interlacing of the 28-day cured samples exhibited greater complexity. This was characterized by an increased presence of needle-like calcium aluminate silicate hydrate (C-A-S-H) gel, the same results as in previous reports [32,33], in comparison to the 7-day cured samples. This increase in structural complexity markedly enhances the mechanical properties of the Yellow River sediment-based flood-prevention stones after 28 days of standard curing, thereby corroborating our hypotheses presented in the preceding discussion. 

Figure 9a–e (left column) illustrate the SEM images of the Yellow River sediment-based flood-prevention stones that have undergone a standard 7-day curing process. The results demonstrated the formation of C-A-S-H gel within the specimens during a relatively short curing period. The structure of the C-A-S-H gel continued to expand as the slag content increased, particularly when the dosage of slag in the specimen was below 8%. However, when the slag content exceeded 8% of the total mass of the specimen, the structure of C-A-S-H gels in the specimen was significantly reduced, accompanied by a large number of unreacted spherical slag particles. This structural alteration led to a modification in the compressive strength of the specimen, which aligned with the results of our prior experimental work (Figure 5).

In contrast, Figure 9f–j (right column) show the SEM images of the 28-day cured stones. Following an extended curing period, the images revealed the formation of numerous needle-like C-A-S-H gels, which significantly contributed to the observed increase in the compressive strength of the specimens. When the slag content was increased above an 8% mass fraction, the SEM images show that the observed hydration products were intricately interwoven to form a stable structure. However, at lower slag contents (below 6%), the porous hydration products resulted in a decrease in the compressive properties of the specimens (Figure 9f,g). This observed trend also corroborated the results obtained during the compressive strength testing discussed in the previous section.

#### 3.3.3. XRD Analysis for Composition of Flood-Prevention Stones

**Figure 10:**
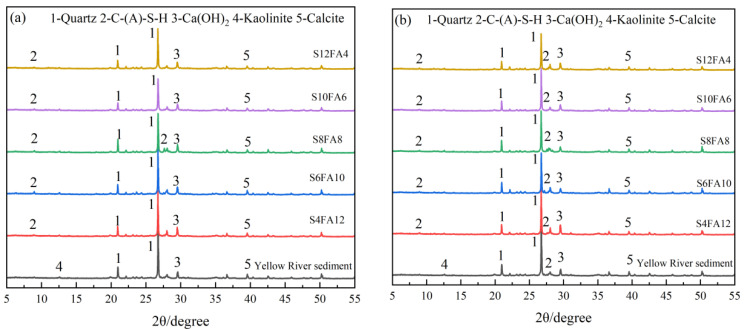
XRD testing of prepared flood-prevention stones after 7 and 28 days of curing. (**a**) XRD testing after 7 days of curing. (**b**) XRD testing after 28 days of curing.

The X-ray diffraction (XRD) analysis results for the Yellow River sediment-based flood-prevention stones, which were cured for 7 and 28 days, are illustrated in Figure 10. The raw sediment from Sanmenxia was found to consist of calcite, montmorillonite, and a significant amount of quartz [34,35,36]. As depicted in Figure 10a, all specimens prepared from Yellow River sediment exhibited signals indicative of C-A-S-H gel formation during testing. Additionally, a notable reduction in the intensity of the quartz peak was observed in the XRD tests conducted under a curing condition of 7 days. The results indicated that the SiO_2_ present in the raw material participated in the hydration reaction, contributing to the formation of a gel with enhanced compressive strength. It is noteworthy that the specimen labeled S8FA8, which displayed the most pronounced C-A-S-H gel signal in the XRD spectrum, exhibited superior performance during the compressive strength tests (Figure 5).

Similarly, the specimens that underwent a curing time of 28 days yielded identical results (Figure 10b). The stronger C-A-S-H gel signal observed in the XRD test implied that the production of the gel should be promoted after prolonging the curing time, which indicated an enhancement in the compressive strength of the specimens. This conclusion was corroborated by the results of the preceding compressive strength tests and the SEM images.

### 3.4. Environmental and Economic Evaluations

The quantity of each component employed in the binder during the solidification of Yellow River sediment had a direct influence on the cost and CO_2_ emissions associated with the manufacturing process of artificial flood-prevention stones. Indeed, these indices exerted a considerable influence on the practical utility of the prepared stones. In this study, we conducted an evaluation of the CO_2_ emissions and manufacturing costs associated with the production of our testing specimens.

#### 3.4.1. Environmental Evaluation

**Figure 11:**
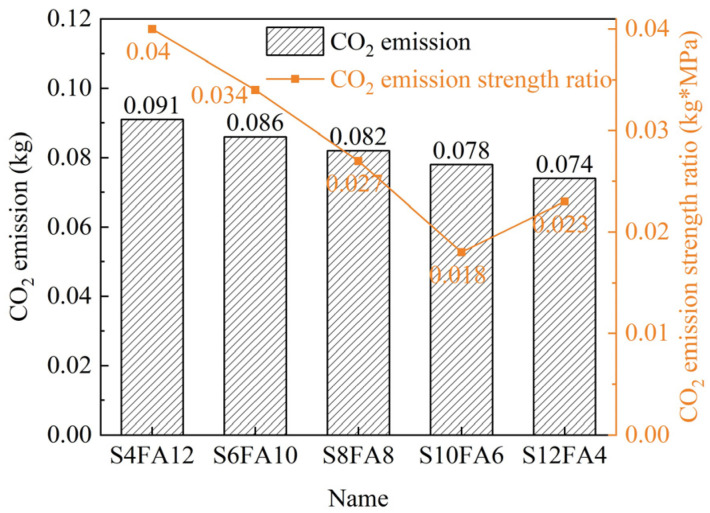
Carbon emissions calculation on Yellow River sediment-based flood-prevention stones.

In evaluating the environmental impact of the artificial flood-prevention stones, we identified the ratio of CO_2_ emissions to the corresponding intensity as a key evaluation index. The corresponding calculation data are presented in Figure 11. S10FA6 exhibited the lowest emissions of 0.18 kg (CO_2_)/(kg·MPa) due to its satisfactory compression strength, demonstrating minimal environmental impact.

#### 3.4.2. Economic Analysis

**Figure 12:**
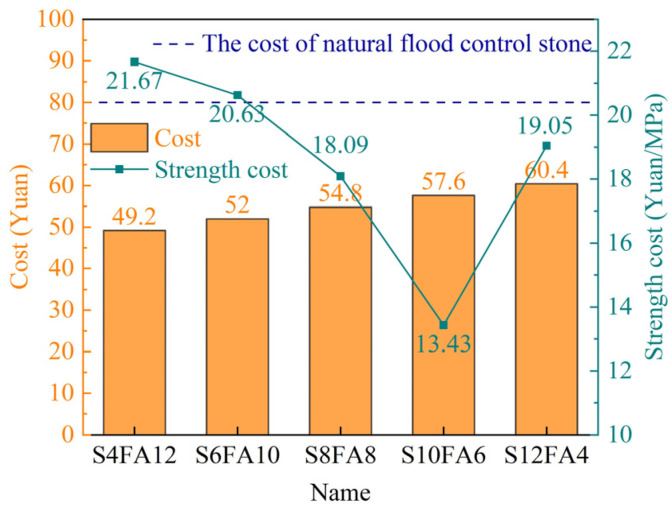
Cost of strength for Yellow River sediment-based flood-prevention stones.

In this section, we set out to examine the production costs of artificial flood-prevention stones through two scenarios. The total production costs of the prepared stones were evaluated (Figure 12). As the proportion of higher-priced slag in the binder increases, the cost of the artificial stones generally rises. The specimen labelled as S12F4 exhibited the highest production cost at 60.4 yuan/t (8.3 USD/t), which was significantly lower than the price of commercially available natural flood-prevention stones, which is approximately 80 yuan/t (11 USD/t).

Additionally, the ratio of cost to compressive strength was employed as an additional reference indicator. With the assistance of the higher compressive strength, the lowest calculated value, 13.4 yuan/t·MPa (1.4 USD/t·MPa), was obtained for the specimen designated as S10F6. These favorable outcomes instill confidence in the prospective utilization of the prepared artificial flood-prevention stones.

## 4. Summary and Conclusions

In this study, we have employed slag, fly ash, and quicklime as binders with a 20% additive concentration to prepare a novel artificial Yellow River sediment-based flood-prevention stone. The main work of our study is outlined below:

1. Cross-verifying the theoretical and experimental results on compressive strength. The results of an orthogonal experimental design, in conjunction with Design-Expert for simulation prediction, were employed to ensure the compressive strength of a prepared artificial Yellow River sediment-based flood-prevention stone with a high degree of accuracy. Furthermore, the individual components of the binder that influence the compressive strength under different curing durations have been elucidated.
2. Clarification of the mechanism of high compressive strength of prepared specimens. Analyses conducted using scanning electron microscopy (SEM) and X-ray diffraction (XRD) revealed the formation of C-A-S-H gel and its growth at different curing periods.
3. Demonstration of the environmental friendliness and economy of artificial Yellow River sediment-based flood-prevention stones. The CO_2_ emissions associated with the preparation of the artificial flood-prevention stones were calculated. In accordance with the optimal compressive strength conditions, the emission on preparation of the desired stones was determined to be 0.18 kg (CO_2_)/(kg·MPa), which is considered to be typical of environmentally friendly production processes.

Based on the research findings, we have derived the following relevant conclusions:

1. Following the completion of the standard curing procedure over a 28-day period, the compressive strength of the prepared artificial stone was found to be 4.29 MPa. The pH of the identical specimen leachate was found to stabilize at 8.2 after 30 h of immersion. The softening coefficient of the S10FA6 specimen was tested with a value of over 0.7 under wet conditions. These important results confirmed that the prepared stones with the selected binder met the specifications for artificial flood-prevention stones.
2. The pozzolanic reaction between CaO in slag and active SiO_2_ and Al_2_O_3_ in fly ash to form a C-A-S-H gel was proved as the main reason for the compressive strength enhancement of the specimens. The testing results on SEM and XRD of the tested specimens demonstrated a direct correlation between the alterations in the structure of the C-A-S-H gel and the compressive strength of the blocks.
3. It has been determined that the cost of the produced artificial flood-prevention stones, utilizing various binders, is notably lower than the market price for commercially available natural stones. The cost-effectiveness of the artificial stones will facilitate their promotion in the market.

The potential applications of solidified Yellow River sediment in other areas of construction and engineering are currently under investigation in our laboratory.

## Figures and Tables

**Table 1:** Chemical composition of Yellow River sediment, slag and fly ash.

Chemical Composition (%)	Yellow River Sediment	Slag	Fly Ash
CaO	11.09	49.42	7.20
SiO_2_	57.02	25.57	46.44
Fe_2_O_3_	6.47	0.31	3.12
Al_2_O_3_	15.88	13.58	38.01
TiO_2_	0.90	2.15	0.61
Na_2_O	1.17	0.44	0.33
SO_3_	0.15	2.36	0.69
K_2_O	3.31	0.33	0.88
MgO	3.30	5.32	0.23
Other	0.71	0.52	2.19

**Table 2:** Dosage of each substance in the binder *^a^*.

Entry	Slag (%)	Fly Ash (%)	Quicklime (%)
1	2	2	16
2	2	5	13
3	2	8	10
4	5	2	13
5	5	5	10
6	5	8	7
7	8	2	10
8	8	5	7
9	8	8	4

*^a^* The Yellow River sediment accounts for 80% of the total mass of the specimens.

**Table 3:** Design of slag replacement fly ash in single-factor influence experiment *^a^*.

Name	Slag (%)	Fly Ash (%)	Quicklime (%)
S4FA12	4	12	4
S6FA10	6	10	4
S8FA8	8	8	4
S10FA6	10	6	4
S12FA4	12	4	4

*^a^* The Yellow River sediment accounts for 80% of the total mass of the specimens.

## Data Availability

The original contributions presented in the study are included in the article, further inquiries can be directed to the corresponding authors.
